# Environmental and Clinical Determinants of Vitamin D Status in Breast Cancer Patients: A Multivariable Analysis

**DOI:** 10.3390/nu18152512

**Published:** 2026-08-03

**Authors:** Dorota Weber, Robert Łuczyk, Anna Pacian, Teresa Kulik, Monika Baryła-Matejczuk

**Affiliations:** 1 Faculty of Health Sciences, WSEI University, 20-209 Lublin, Poland; teresa.kulik@gmail.com; 2 Faculty of Medical and Health Sciences, University of Siedlce, 08-110 Siedlce, Poland; luczykrobert@op.pl; 3 Department of Health Education, Faculty of Nursing Development, Medical University in Lublin, 20-059 Lublin, Poland; anna.pacian@umlub.edu.pl; 4 Institute of Psychology and Human Sciences, WSEI University, 20-209 Lublin, Poland; monika.baryla@wsei.pl

**Keywords:** vitamin D, 25-hydroxyvitamin D, breast cancer, molecular subtypes, dietary patterns, psychological distress, BMI, sleep quality, pHDI-10, Distress Thermometer

## Abstract

Background/Objectives: Vitamin D deficiency is commonly observed in patients with breast cancer and may affect disease course and treatment outcomes. The determinants of vitamin D status in this population remain incompletely understood, particularly with respect to lifestyle, psychosocial, and tumour-related factors. The aim of this study was to identify environmental and clinical factors associated with serum 25-hydroxyvitamin D [25(OH)D] concentrations in women with breast cancer, with particular emphasis on dietary patterns, psychological distress, sleep quality, and tumour molecular subtype. Methods: A cross-sectional observational study was conducted among 101 women with histopathologically confirmed invasive breast cancer, recruited at the St. John of Dukla Oncology Centre of the Lublin Region (COZL) in Lublin, Poland, between 2018 and 2019. Serum 25(OH)D concentrations were measured by electrochemiluminescence immunoassay (ECLIA; Roche Diagnostics) within 2–8 weeks after surgical treatment. Clinical and lifestyle data were collected using standardised questionnaires and medical records. Dietary patterns were assessed with the KomPAN questionnaire (pro-healthy diet index pHDI-10 and non-healthy diet index nHDI-14). Psychological distress was measured with the Distress Thermometer. Multiple linear regression with backward stepwise elimination was applied to identify factors independently associated with of 25(OH)D concentration. Results: Insufficient vitamin D status (25(OH)D < 30 ng/mL) was found in 56.4% of participants. The multivariable regression model was statistically significant (F(6,92) = 15.298; *p* < 0.001), explaining 49.9% of the variance in 25(OH)D concentrations. Factors independently associated with lower 25(OH)D included higher BMI (b = −0.715; *p* = 0.005), sleep disturbances (b = −6.257; *p* = 0.020), higher psychological distress score (b = −2.263; *p* < 0.001), and luminal B versus luminal A subtype (b = −5.909; *p* = 0.025). A higher pro healthy diet index (pHDI-10) was independently associated with higher 25(OH)D (b = 0.245; *p* = 0.040). Conclusions: Vitamin D status in patients with breast cancer is shaped by complex interactions among modifiable lifestyle factors and tumour characteristics. Targeted interventions addressing diet quality, psychological well-being, sleep health, and weight management may improve vitamin D status in this population. The association between molecular subtype and 25(OH)D concentrations warrants further prospective investigation. These findings should be interpreted with caution, as vitamin D supplementation and direct measures of sun exposure—both established determinants of 25(OH)D—could not be included as covariates and may account, at least in part, for the associations reported.

## 1. Introduction

Breast cancer remains the most frequently diagnosed malignancy and a leading cause of cancer-related death among women worldwide [1]. Identification of modifiable risk and prognostic factors is therefore of primary importance for both prevention and clinical management. Vitamin D—a lipophilic secosteroid hormone with pleiotropic biological functions—has attracted considerable scientific interest due to its potential role in breast cancer biology. Beyond its well-documented role in calcium homeostasis and bone metabolism, vitamin D exerts antiproliferative, pro-apoptotic, anti-angiogenic, and immunomodulatory effects that may be relevant to tumour initiation and progression [2,3]. Epidemiological studies have demonstrated associations between lower serum 25 hydroxyvitamin D [25(OH)D] concentrations—the principal circulating form and the recognised biomarker of vitamin D status—and increased breast cancer risk, more aggressive tumour phenotypes, and poorer clinical outcomes [4,5,6]. Notably, vitamin D deficiency is particularly prevalent among oncological patients: data from various populations indicate that 40–80% of women with breast cancer exhibit suboptimal 25(OH)D concentrations at the time of diagnosis [7,8]. Despite this, the determinants of vitamin D status in the breast cancer population remain incompletely characterised. Whereas classical determinants such as sun exposure, dietary intake, body mass index (BMI), and age are well established in the general population [9], the relative contribution of tumour-related and psychosocial factors in oncological patients is less well understood. Tumour molecular subtype—classified on the basis of hormone receptor expression (oestrogen receptor [ER], progesterone receptor [PR]), HER2 receptor, and proliferative index Ki-67—represents a particularly important clinical variable, given evidence suggesting that hormonal signalling may modulate vitamin D metabolism [10,11]. Furthermore, emerging data indicate that psychological distress, sleep quality, and dietary patterns may independently influence vitamin D status; however, these factors have rarely been assessed simultaneously in a multivariable model in patients with breast cancer [12,13]. The primary objective of this study was to identify environmental and clinical determinants of serum 25(OH)D concentrations in women with histopathologically confirmed invasive breast cancer using multiple regression analysis. Secondary objectives included describing the distribution of vitamin D status by molecular subtype and the prevalence of vitamin D deficiency in the study sample.

## 2. Materials and Methods

### 2.1. Study Design and Setting

A cross-sectional observational study was conducted at the St. John of Dukla Oncology Centre of the Lublin Region (COZL) in Lublin, Poland. Patient recruitment took place between 2018 and 2019.

### 2.2. Ethical Considerations

The study protocol was approved by the Bioethics Committee of the St. John of Dukla Oncology Centre of the Lublin Region (COZL) in Lublin, Poland (Resolution No. KE-0254/78/2019; approval date 28 February 2019), and the study was conducted in accordance with the principles of the Declaration of Helsinki [14]. Written informed consent was obtained from all participants prior to enrolment.

### 2.3. Study Participants

Women were eligible if they met all of the following inclusion criteria: (1) histopathologically confirmed invasive breast cancer; (2) completion of surgical treatment; (3) availability of molecular subtype classification based on immunohistochemical (IHC) examination; and (4) age ≥ 18 years. No exclusion criteria related to vitamin D supplementation or comorbidities were applied, as the study aimed to reflect real-world clinical conditions. A total of 101 patients were recruited by consecutive sampling.

### 2.4. Blood Sampling and Biochemical Analysis

Venous blood samples were collected within 2–8 weeks after surgical treatment during standard follow-up visits. All samples were drawn in the morning (08:00–11:00) under fasting conditions to minimise diurnal variability. Samples were processed within 2 h of collection and stored until analysis. Serum 25(OH)D concentrations were measured by electrochemiluminescence immunoassay (ECLIA; Vitamin D Total Test, Roche Diagnostics, Mannheim, Germany) on a Cobas e410 analyser (Roche Diagnostics, Mannheim, Germany). The assay measured total 25-hydroxyvitamin D [25(OH)D + _2 3_ 25(OH)D]. Analytical sensitivity was 3.0 ng/mL; functional sensitivity (CV = 20%) was 7.5 ng/mL. Within-assay and between-assay coefficients of variation were.

### 2.5. Molecular Subtype Classification

Molecular subtypes were determined by immunohistochemistry (IHC) for oestrogen receptor (ER), progesterone receptor (PR), HER2 receptor, and proliferative index Ki-67, performed on formalin-fixed, paraffin-embedded (FFPE) tissue. For equivocal IHC results for HER2 (score 2+), HER2 status was confirmed by fluorescence in situ hybridisation (FISH). Hormone receptor expression was defined as positive at ≥1% of tumour cell nuclei staining positive, in accordance with ASCO/CAP guidelines [15]. Subtypes were classified as follows: luminal A (ER+ and/or PR+, HER2−, Ki-67 < 20%), luminal B (ER+ and/or PR+, HER2−, Ki-67 ≥ 20%), HER2-positive (ER−, PR−, HER2+), and triple negative (ER−, PR−, HER2−).

### 2.6. Anthropometric and Clinical Data

Body mass index (BMI; kg/m^2^) was calculated as body weight (kg) divided by height squared (m^2^) and categorised according to WHO criteria: normal weight (18.5–24.9), overweight (25.0–29.9), and obesity (≥30.0). Menopausal status was defined as the absence of menstruation for ≥12 consecutive months or prior bilateral oophorectomy. Season of blood collection was dichotomised into Summer/Autumn (April–September) and Winter/Spring (October–March). Outdoor physical activity and sleep problems (difficulties falling asleep, nocturnal awakenings, or excessive sleep duration) were assessed by a self-report questionnaire.

### 2.7. Dietary Assessment

Dietary habits were assessed using the KomPAN questionnaire—a validated food frequency questionnaire developed for Polish adult populations. The pro-healthy diet index (pHDI-10), encompassing 10 food groups characteristic of a health-promoting dietary pattern (including vegetables, fruits, whole-grain products, fish, and legumes), and the non-healthy diet index (nHDI-14), based on 14 food groups associated with adverse health outcomes (including processed meat, fast food, and sugar-sweetened beverages), were calculated according to the established scoring methodology [16].

### 2.8. Psychological Assessment

Psychological distress was assessed using the Distress Thermometer (DT)—a single-item visual analogue scale ranging from 0 (no distress) to 10 (extreme distress), recommended by the National Comprehensive Cancer Network (NCCN) in oncological settings [17]. A score ≥ 4 was considered indicative of clinically significant distress.

### 2.9. Statistical Analysis

Statistical analyses were performed using Statistica 14 (Cloud Software Group, Inc., Fort Lauderdale, FL, USA, 2023). Descriptive statistics included frequencies, percentages (%), means ± standard deviations (SD), medians with interquartile ranges (IQR: Q1–Q3), and range (minimum–maximum). Normality of variable distributions was assessed using the Shapiro–Wilk test. Given significant departure of 25(OH)D concentrations from normality (*p* < 0.05), differences in vitamin D levels across molecular subtypes were examined using the Kruskal–Wallis test, with pairwise comparisons performed using Dunn’s test. Multiple linear regression with backward stepwise elimination was used to identify predictors of 25(OH)D concentration. Ten candidate variables were entered into the initial model: BMI, age, menopausal status, season of blood collection, sleep problems, outdoor physical activity, molecular subtype, Distress Thermometer score, pHDI-10, and nHDI-14. Variables were retained or removed based on the F statistic: inclusion required F ≥ 4, and removal occurred at F < 3 (corresponding approximately to significance levels of *p* = 0.05 and *p* = 0.10). The procedure was terminated when no variable met the removal criterion. Model assumptions were verified as follows. Residual independence was assessed using the Durbin–Watson statistic (d = 2.08; serial correlation r = −0.042), indicating no substantial autocorrelation. Residual normality was evaluated graphically using a quantile–quantile (Q–Q) plot, which indicated acceptable fulfilment of this assumption with minor deviations at the tails. Homoscedasticity and linearity were assessed from plots of residuals versus predicted values, with no systematic patterns observed. Collinearity was assessed using tolerance values, which ranged from 0.77 to 0.97 for all predictors, excluding substantial multicollinearity. A significance level of α = 0.05 was adopted. Two participants with incomplete covariate data were excluded from the regression model on a listwise basis (complete-case analysis); no imputation was performed. As this study represents a secondary analysis of an existing dataset, no a priori sample size calculation had been performed; a post hoc power analysis was therefore conducted for the primary comparison of serum 25(OH)D concentrations across molecular subtypes, based on the observed effect size.

## 3. Results

### 3.1. Sample Characteristics

A total of 101 women with invasive breast cancer were enrolled. Participant age ranged from 32 to 71 years, with a mean of 52.55 ± 9.50 years. The majority were postmenopausal (59.41%). More than half had excess body weight (BMI ≥ 25.0 kg/m^2^; 57.43%), including 17.83% with obesity. Nearly one third reported sleep disturbances (31.68%), and fewer than half reported regular outdoor physical activity (44.55%). The most frequent molecular subtypes were luminal A (40.59%) and luminal B (31.68%), followed by triple-negative (16.83%) and HER2-positive (10.89%). Blood samples were collected slightly more often during Summer/Autumn than Winter/Spring (57.43% vs. 42.57%). Full demographic and clinical characteristics are presented in Table 1.

### 3.2. Dietary and Psychological Characteristics

The mean Distress Thermometer score was 4.46 ± 2.74 (median: 4.0; IQR: 2.0–6.0), indicating a clinically significant level of distress in the study group. The mean pro-healthy diet index (pHDI-10) was 27.50 ± 9.96 (median: 26.70; IQR: 20.20–34.60), and the mean non-healthy diet index (nHDI-14) was 13.40 ± 6.98 (median: 13.21; IQR: 8.21–16.57). These parameters are summarised in Table 2.

### 3.3. Vitamin D Status by Molecular Subtype

Overall, 57 of 101 patients (56.44%) had 25(OH)D concentrations below 30 ng/mL. The median serum 25(OH)D for the entire study sample was 26.0 ng/mL (IQR: 18.0–36.0 ng/mL). Statistically significant differences in 25(OH)D concentrations were found across molecular subtypes (Kruskal–Wallis H(3, *n* = 101) = 9.001; *p* = 0.029). The highest median values were observed in the HER2-positive subtype (39.0 ng/mL; IQR: 27.0–45.0) and luminal A (30.0 ng/mL; IQR: 21.0–39.0), whereas the lowest were in the triple-negative (20.0 ng/mL; IQR: 15.0–31.0) and luminal B (24.0 ng/mL; IQR: 18.5–31.5) subtypes. Pairwise comparisons using Dunn’s test did not reveal statistically significant differences between individual subtype pairs, suggesting a global rather than pairwise distributional shift. A post hoc power analysis for this comparison indicated a small effect size (ε^2^ = 0.062), corresponding to an achieved statistical power of 54.8% (α = 0.05), suggesting that the study may have been underpowered to detect small-to-moderate differences between molecular subtypes. The proportion of patients with 25(OH)D < 30 ng/mL was highest in the luminal B (65.63%) and triple negative (64.71%) subtypes. Detailed results are presented in Table 3.

### 3.4. Multivariable Predictors of Vitamin D Status

The multiple linear regression model with backward stepwise elimination retained six predictors and was statistically significant (F(6,92) = 15.298; *p* < 0.001), explaining 49.9% of the variance in 25(OH)D concentrations (R^2^ = 0.499; adjusted R^2^ = 0.467). The standard error of the estimate was 10.900 ng/mL. Results are presented in Table 4.

Higher 25(OH)D concentrations were independently associated with lower BMI (b = −0.715; *p* = 0.005), absence of sleep disturbances (b = −6.257; *p* = 0.020), higher pHDI-10 score (b = 0.245; *p* = 0.040), and lower psychological distress (b = −2.263; *p* < 0.001). Patients with luminal B breast cancer had significantly lower 25(OH)D compared with luminal A (b = −5.909; *p* = 0.025) after adjustment for all other predictors. The difference between triple-negative and luminal A showed a similar trend but did not reach significance (b = −5.468; *p* = 0.083).

Model diagnostics confirmed adequate fulfilment of regression assumptions. The Durbin–Watson statistic (d = 2.08) indicated absence of residual autocorrelation. Tolerance values for all predictors ranged from 0.77 to 0.97, excluding substantial multicollinearity.

## 4. Discussion

The principal findings of this study are threefold. First, vitamin D deficiency—defined as 25(OH)D < 30 ng/mL—was highly prevalent, affecting more than half (56.4%) of the patients with breast cancer studied. Second, serum 25(OH)D concentrations differed significantly across molecular subtypes, with the lowest values in triple-negative and luminal B tumours. Third, a multivariable model incorporating environmental and clinical variables explained approximately 50% of the variance in 25(OH)D concentrations, identifying BMI, psychological distress, sleep quality, pro-healthy dietary patterns, and molecular subtype as independent determinants. Because several established determinants of 25(OH)D status—most notably vitamin D supplementation and directly measured sun exposure—could not be entered into the multivariable model, these findings should be regarded as descriptive, hypothesis-generating associations rather than evidence of independent, unconfounded effects; residual confounding cannot be excluded and is discussed further below.

The high prevalence of vitamin D deficiency observed in our study sample is consistent with data from other European and Polish breast cancer populations [6,18]. Several mechanisms may account for the particular susceptibility of oncological patients to hypovitaminosis D, including reduced sun exposure due to treatment-related fatigue, impaired hepatic and renal metabolism secondary to tumour-associated inflammation, and increased sequestration of 25(OH)D in adipose tissue [3,19].

The association between higher BMI and lower 25(OH)D concentrations, confirmed in our multivariable model, is well established and most likely reflects volumetric dilution of vitamin D in adipose tissue and reduced bioavailability of this lipophilic vitamin [20]. A substantial proportion of our participants (57.4%) were overweight or obese, which itself may constitute an independent prognostic factor for cancer and warrants clinical attention beyond its impact on vitamin D status. The independent association between psychological distress and lower 25(OH)D concentrations is a novel and clinically relevant finding. Although the biological mechanisms underlying this relationship require further elucidation, several pathways have been proposed. Chronic psychological stress activates the hypothalamic–pituitary adrenal (HPA) axis, leading to elevated cortisol levels, which may inhibit hepatic 25 hydroxylase activity and thereby reduce 25(OH)D synthesis [21]. Conversely, vitamin D itself may exert neuroprotective and immunomodulatory effects that attenuate the neuroendocrine stress response, suggesting a potential bidirectional relationship [22]. The mean Distress Thermometer score in our study sample (4.46 ± 2.74) exceeded the clinically significant threshold of 4.0, underscoring the high psychological distress burden in this population. Nonetheless, because vitamin D supplementation and comorbidity status were not recorded, we cannot exclude that part of the BMI and distress associations reflects unmeasured confounding rather than a direct effect of adiposity or psychological distress on 25(OH)D metabolism.

Sleep disturbances were independently associated with lower 25(OH)D concentrations after multivariable adjustment. This relationship may be mediated by disruption of the circadian pineal–melatonin axis, which has documented interactions with vitamin D receptor (VDR) signalling [23]. Alternatively, sleep problems may serve as a proxy indicator of generally poor health status and reduced outdoor activity, indirectly limiting cutaneous vitamin D synthesis. Nearly one third of our participants reported sleep disturbances, a proportion consistent with published rates in oncological populations [24]. As sleep quality was captured with a single non-validated item and supplementation status was unavailable, this association should be regarded as preliminary rather than conclusive.

The positive association between pHDI-10 and 25(OH)D concentrations supports the role of dietary patterns in modulating vitamin D status. Foods contributing to higher pHDI-10 scores—including oily fish, eggs, and vitamin D–fortified dairy products—constitute the principal dietary sources of vitamin D [25]. The effect size, although statistically significant, was modest (b = 0.245 per unit pHDI-10), consistent with the generally limited contribution of diet to overall vitamin D status relative to cutaneous synthesis and supplementation [9]. Because supplement use could not be captured and may co-vary with a health-conscious diet, the modest association reported here may partly reflect unmeasured confounding by supplementation or sun-exposure behaviours rather than diet per se.

The association between molecular subtype and 25(OH)D concentrations observed in our study is biologically plausible and aligns with a growing body of evidence. Luminal B and triple-negative subtypes, characterised respectively by higher proliferative activity and absence of hormone receptor signalling, were associated with lower 25(OH)D concentrations in our study sample. Oestrogen receptor signalling has been shown to upregulate the expression of vitamin D–metabolising enzymes and VDR in breast epithelial cells [10], suggesting that ER-negative tumours may be associated with impaired local vitamin D metabolism. Additionally, systemic inflammation—more pronounced in aggressive molecular subtypes—may accelerate 25(OH)D catabolism through induction of CYP24A1 [26,27,28,29,30,31]. It must be emphasised that this association was estimated without adjustment for vitamin D supplementation, comorbidities, or directly measured sun exposure, any of which may differ systematically across molecular subtypes (for example, through differences in treatment regimens or follow-up intensity); the possibility that this finding reflects confounding rather than a direct tumour-driven effect cannot be excluded and requires prospective confirmation with fuller covariate adjustment.

Several limitations of this study warrant acknowledgement. The cross-sectional design precludes causal inference; temporal relationships between the identified determinants and 25(OH)D status cannot be established. The sample size, although adequate for the regression model, limits statistical power for pairwise post hoc comparisons across molecular subtypes, particularly for the HER2-positive subgroup (*n* = 11), and increases the risk of a type II error in these secondary, subtype-level comparisons. Vitamin D supplementation status was not systematically recorded as a covariate, representing a potential source of unmeasured confounding because supplementation tends to raise 25(OH)D concentrations, its omission may have attenuated or otherwise biased the associations reported here, and a sensitivity analysis excluding supplement users could not be performed. Moreover, outdoor sun exposure—the principal determinant of vitamin D status—was captured only dichotomously as self-reported outdoor physical activity, which may not fully reflect UVB radiation dose: information on time spent outdoors, skin area exposed, sunscreen use, and clothing habits was not collected. Comorbidities and concomitant medication use, which may independently affect 25(OH)D metabolism, were not entered as covariates in the multivariable model and could represent additional unmeasured confounders; age, menopausal status, and season of blood collection were tested as candidate predictors but were not retained by the backward elimination procedure. Sleep problems and outdoor physical activity were assessed using non-validated, single-item self-report questions rather than a psychometrically validated instrument such as the Pittsburgh Sleep Quality Index, which may have introduced measurement error. Serum 25(OH)D was measured within a 2–8 week postoperative window, and the exact interval between surgery and blood sampling, together with the timing of adjuvant treatment initiation, was not modelled as a covariate and may have introduced additional clinical heterogeneity. The pHDI-10 captures overall diet quality rather than vitamin D intake specifically, and serum calcium, parathyroid hormone, and vitamin D-binding protein were not measured. The use of stepwise variable selection, while standard in exploratory analyses of this size, can yield optimistic standard errors and should be interpreted with corresponding caution. Finally, the study was conducted at a single centre in Poland, which may limit generalisability to populations with different ethnic characteristics, latitudes, or healthcare practices.

Future prospective studies with larger, multicentre samples should systematically record vitamin D supplementation, direct measures of sun exposure, comorbidities, and time from surgery to sampling, and should employ validated instruments for sleep quality. Repeated 25(OH)D measurements, intervention trials of vitamin D correction, and exploration of vitamin D receptor polymorphisms and supplementation effects by molecular subtype would help clarify whether the associations identified here are amenable to clinical modification.

## 5. Conclusions

This study demonstrates that vitamin D deficiency is highly prevalent among patients with breast cancer and is associated with a multifactorial interplay of modifiable lifestyle factors and tumour characteristics. BMI, psychological distress, sleep disturbances, and diet quality were independently associated with 25(OH)D concentrations, accounting for approximately half of the observed variability. These associations should be interpreted cautiously: key determinants of 25(OH)D status, particularly vitamin D supplementation and directly measured sun exposure, were not available for adjustment, and residual confounding cannot be excluded. The association of luminal B molecular subtype with lower vitamin D status additionally suggests a biologically tumour-related dimension of vitamin D metabolism in this population. These findings support the integration of vitamin D status assessment and targeted lifestyle counselling into routine oncological care for patients with breast cancer. Multidisciplinary interventions addressing nutritional quality, psychological well-being, sleep health, and weight management may collectively improve vitamin D status and potentially contribute to favourable treatment outcomes. Prospective studies with larger sample sizes, standardised supplementation protocols, and comprehensive sun exposure data are needed to confirm the causal significance of these associations and to evaluate the clinical impact of correcting vitamin D deficiency in breast cancer care.

## Figures and Tables

**Table 1:** Demographic and clinical characteristics of study participants (*n* = 101). M, mean; SD, standard deviation.

**Variable**		**M**	**SD**
Age (years)		52.55	9.50
BMI (kg/m^2^)		26.54	4.61
**Variable**	**Category**	***n***	**%**
BMI category	Normal weight (18.5–24.9)	43	42.57
	Overweight (25.0–29.9)	40	39.60
	Obesity (≥30.0)	18	17.83
Menopausal status	Premenopausal	41	40.59
	Postmenopausal	60	59.41
Season of blood collection	Winter/Spring (October–March)	43	42.57
	Summer/Autumn (April–September)	58	57.43
Outdoor physical activity	Yes	45	44.55
	No	56	55.45
Sleep disturbances	Yes	32	31.68
	No	69	68.32
Molecular subtype	Luminal A	41	40.59
	Luminal B	32	31.68
	HER2-positive	11	10.89
	Triple-negative	17	16.83

**Table 2:** Distress Thermometer, pro-healthy diet index (pHDI-10), and non-healthy diet index (nHDI-14) (*n* = 101). M, mean; SD, standard deviation. For the Distress Thermometer, *n* = 99 (two participants did not report their distress level).

Variable	M	SD	Median	Q1	Q3	Min	Max
Distress Thermometer	4.46	2.74	4.0	2.0	6.0	0	10
pHDI-10	27.50	9.96	26.70	20.20	34.60	8.20	47.0
nHDI-14	13.40	6.98	13.21	8.21	16.57	0.0	30.21

**Table 3:** Vitamin D status [25(OH)D] by breast cancer molecular subtype (*n* = 101).

Molecular Subtype	*n*	Median 25(OH)D (ng/mL)	IQR (Q1–Q3)	25(OH)D < 30 ng/mL, *n* (%)
Luminal A	41	30.0	21.0–39.0	20 (48.78%)
Luminal B	32	24.0	18.5–31.5	21 (65.63%)
HER2-positive	11	39.0	27.0–45.0	5 (45.45%)
Triple-negative	17	20.0	15.0–31.0	11 (64.71%)
Total	101	26.0	18.0–36.0	57 (56.44%)

Kruskal–Wallis test: H(3, *n* = 101) = 9.001; *p* = 0.029. IQR, interquartile range (Q1–Q3).

**Table 4:** Multiple linear regression model of predictors of serum 25(OH)D concentration in patients with breast cancer (*n* = 99).

Predictor	β*	SE β*	b	SE b	t	*p*-Value (95% CI for b)
Intercept	—	—	56.518	7.525	7.511	<0.001 (41.57, 71.46)
Mol. subtype: Luminal B vs. Luminal A	−0.185	0.081	−5.909	2.587	−2.284	0.025 (−11.05, −0.77)
Mol. subtype: Triple-negative vs. Luminal A	−0.139	0.079	−5.468	3.116	−1.755	0.083 (−11.66, 0.72)
BMI (kg/m^2^)	−0.223	0.078	−0.715	0.250	−2.859	0.005 (−1.21, −0.22)
Sleep disturbances (yes vs. no)	−0.195	0.083	−6.257	2.645	−2.366	0.020 (−11.51, −1.00)
pHDI-10	0.164	0.079	0.245	0.117	2.086	0.040 (0.01, 0.48)
Distress Thermometer	−0.416	0.084	−2.263	0.456	−4.962	<0.001 (−3.17, −1.36)

R = 0.707; R^2^ = 0.499; adjusted R^2^ = 0.467; F(6,92) = 15.298; *p* < 0.001; SE of estimate: 10.900; *n* = 99. β*, standardised regression coefficient; b, unstandardised regression coefficient; SE, standard error; pHDI-10, Pro-healthy Diet Index-10. Reference category: Luminal A. Two patients excluded due to missing data. 95% confidence intervals for b were calculated as b ± t(0.025,92) × SE b (t(0.025,92) = 1.986).

## Data Availability

The data presented in this study are available on request from the corresponding author. The data are not publicly available due to institutional data protection regulations.
